# Immediate mood changes and practice adherence during a self-directed SKT1 meditation program in university students: An intensive longitudinal study

**DOI:** 10.1371/journal.pone.0350556

**Published:** 2026-05-29

**Authors:** Sorachai Kamollimsakul, Suparpit Maneesakorn von Bormann, Somporn Kantharadussadee Triamchaisri

**Affiliations:** 1 Institute of Digital Arts and Science, Suranaree University of Technology, Nakhon Ratchasima, Thailand; 2 Institute of Nursing, Suranaree University of Technology, 111 University Avenue, Suranaree Subdistrict, Muange District, Nakhon Ratchasima, Thailand; 3 SMAEM SKT Meditation Healing Exercise Center; University of Cuu Long, Vietnam; Isha Yoga Centre, INDIA

## Abstract

**Background:**

University students frequently report elevated stress, yet access to formal mental health services is often constrained. Self-directed mind-body practices, such as the Somporn Kantharadussadee Triamchaisri program (SKT1), may offer low-intensity mood management; however, real-world evidence based on repeated within-person assessments remains limited.

**Objectives:**

To examine immediate within-session mood changes, practice adherence, temporal stability, and individual differences associated with self-directed SKT1 practice among university students.

**Methods:**

Twenty-seven university students (*n* = 19 female, 70.37%) completed 28 self-directed SKT1 sessions over 14 days, yielding 710 within-person observations. Negative mood was rated on a 0–10 numeric rating scale (NRS) immediately before and after each session. Data were analyzed using Linear Mixed Models (LMM) with a Diagonal covariance structure, which provided a superior model fit compared to simpler variance components.

**Results:**

Participants demonstrated high engagement, completing a mean of 26.3 sessions (*SD* = 2.8). LMM analysis revealed a highly significant decrease in negative mood immediately following SKT1 practice (*b* = 0.572, *t*(522.79) = 42.12, *p* < .001), representing a large effect size (Paired Cohen’s *d* = 1.00, 95% CI [0.91, 1.09]). These immediate mood changes were consistently observed across all 28 sessions (*p* = .367) and did not vary by practice adherence (*p* = .587). While baseline stress showed a marginal trend toward mood reduction (*b* = −0.128, *p* = .074), baseline anxiety and resilience were not significantly associated with immediate outcomes.

**Conclusions:**

Self-directed SKT1 practice might be associated with consistent, immediate mood reductions that are stable over time and accessible within this specific university sample. These preliminary findings suggest the potential of SKT1 as a feasible ‘per-session’ resource for supporting mood management among students in similar academic contexts. The protocol was registered in the Thai Clinical Trials Registry (TCTR20231016002).

## Introduction

A significant number of university students face mental health challenges. UNESCO reports that 33% of students in 8 countries have mental health issues, mostly late teens [1]. These difficulties frequently disrupt academic performance, with the World Health Organization (WHO) identifying anxiety and depression as leading causes of disability among young adults [2].

Mental health initiatives remain chronically underfunded, often being marginalized within broader public health budgets [2]. This leads to systemic barriers, including staff shortages and prohibitive waiting lists [1]. Critically, intrinsic barriers, such as stigma, prevent students from accessing counseling centers within their universities or local communities [1]. Such constraints highlight the urgent need for autonomous strategies that students can employ independently alongside formal care or in its absence. In this context, self-directed mental health care is no longer merely an optional adjunct but a vital necessity for supporting students’ psychological well-being. Consequently, self-directed interventions that offer flexibility and minimal time commitment, such as meditation, may be particularly suited to the demanding schedules of university students.

Empirical evidence suggests that contemplative practices can support mental health and well-being by reducing stress and enhancing resilience [3–9]. Conventional Mindfulness-Based Programs (MBPs), most notably Mindfulness-Based Stress Reduction (MBSR) or Mindfulness-Based Cognitive Therapy (MBCT), function primarily by anchoring the individual in non-judgmental awareness, leveraging top-down cognitive reappraisal to regulate distress [8,9]. Beyond these mainstream cognitive-based approaches, physiologically oriented interventions offer a distinct pathway to mood regulation. The Somporn Kantharadussadee Triamchaisri meditation (SKT) program is a self-directed meditation modality that has demonstrated encouraging potential in managing psychological outcomes, and is specifically designed to support emotional regulation through structured breathing and mindful attention [10–14]. Operating on a bottom-up paradigm, SKT1 is theorized to leverage structured breathing, which may support autonomic regulation [15]. Theoretically, rather than relying solely on cognitive shifts, SKT is proposed to attenuate sympathetic stress responses and enhance parasympathetic activity, thus distinguishing it from traditional, awareness-based contemplative practices.

Despite its potential, limited research has explored how students engage with SKT1 under minimally prompted, naturalistic conditions, which is a critical step in establishing the ecological validity of the practice. Evaluating SKT1 in naturalistic settings is essential to understand its practical utility beyond controlled clinical environments. Furthermore, whether session-level mood changes persist across repeated practice remains an open question. Based on literature regarding university mental health services, current research frequently relies on single pre–post assessments or short-duration interventions that describe average change at the group level. However, aggregate models often overlook within-person fluctuations and immediate within-session changes because they ignore the nested dependencies inherent in intensive longitudinal data, thus preventing a granular understanding of the dynamic way in which negative mood levels evolve through repeated, self-directed practice. By including a small-scale sample from various academic disciplines (e.g., Engineering, Public Health) and maintaining a neutral researcher-participant dynamic, this exploratory study provides a preliminary look at the feasibility of SKT1 outside of traditional health-related faculties.

The current study adopts a descriptive, observational approach to characterize the immediate within-session mood changes associated with self-directed SKT1 practice. It addresses four primary research questions: (1) whether self-directed SKT1 is associated with immediate within-session reductions in negative mood level (RQ1); (2) whether these immediate changes remain stable across repeated sessions without evidence of incremental accumulation or habituation (RQ2); (3) whether practice adherence is correlated with immediate mood changes (RQ3); and (4) whether baseline levels of stress, anxiety, and resilience predict individual differences in immediate practice-related changes (RQ4).

## Methods

We utilized an intensive longitudinal design to track session-by-session mood changes during 14 days of self-directed SKT1 practice (totaling 28 sessions). Consistent with the study’s focus on intra-individual dynamics rather than causal inference, a control group was omitted to allow for a detailed exploration of within-person mood trajectories. This design specifically addressed the limitations of aggregate pre-post measures by capturing the temporal stability and individual trajectories of mood change. Participants completed 28 self-directed practice sessions. Negative mood was rated immediately before and after each session, and completion was logged via Google Forms. This design focused on within-person change across 710 practice occasions, with each participant serving as their own control. By utilizing repeated assessments at the session level, the study accounted for nested structure of the data (sessions nested within individuals), prioritizing ecological validity and practical feasibility over between-group comparisons, which are more appropriate for later-stage utility trials.

### Participants and recruitment

The study population comprised students from diverse academic disciplines, including Engineering, Public Health, and other faculties. To minimize social desirability and evaluative bias, it is important to note that the primary researcher did not have any teaching or grading responsibilities over the participants. This lack of a pedagogical relationship ensured that participation was voluntary and that responses were not influenced by academic expectations or the teacher-student dynamic.

Participants were 27 university students enrolled at Suranaree University of Technology in North-Eastern Thailand. They were recruited through online and on-campus poster announcements between 9^th^ April 2023 and 4^th^ September 2023. Eligibility criteria included: (1) current university enrollment, (2) age between 18 and 25 years, (3) Thai language fluency, (4) ownership of a smartphone for practice logging, and (5) willingness to engage in daily self-directed practice. Exclusion criteria were: (1) current psychiatric treatment, (2) prior formal meditation training, and (3) severe mental health symptoms requiring immediate clinical intervention.

Participants were drawn from a larger research program examining multiple variables related to SKT1 meditation vs. VR vs. brief CBT. Baseline trait measures of stress, anxiety, and resilience served as between-person (Level-2) predictors in the present analysis; their role as intervention outcomes in a comparative study is reported separately.

### SKT1 meditation program

SKT meditation program is a Thai complementary practice developed by Professor Dr. Somporn Kantharadussadee Triamchaisri (a nurse and neuroscientist), Mahidol University, Thailand since 1978 [16]. The aim of SKT is to promote physiological balance and emotional stability through structured breathing and mindful attention [15,16]. SKT1, the foundational technique, uses nasal inhalation, 3–5 breath hold, and long oral exhalation (20–40 rounds, practiced twice daily). This intentional slow-breathing cycle is theorized to involve vagal stimulation, potentially supporting parasympathetic tone while addressing sympathetic stress responses [16,17]. The approach has been endorsed by the Department of Thai Traditional and Alternative Medicine and has been applied in both clinical and community settings. Previous studies have suggested that SKT meditation techniques may be associated with lower reported stress [10,11,13], reduced anxiety [12,18], and enhanced mental ability [14]. The present study focused exclusively on SKT1, a foundational technique within the SKT system.

### Study procedure

The study was conducted in three sequential phases, designed to support self-directed implementation while capturing real-world mood dynamics.

#### Phase 1: Baseline assessment and SKT1 training.

Participants completed demographic information and baseline measures of resilience, stress, and anxiety. They attended three individual face-to-face sessions (lasting 60 minutes each) conducted by an experienced instructor, which included supportive counseling (40 minutes) and standardized practice instructions (20 minutes). The instructions included: (1) a 2-minute introduction on the theoretical basis of SKT1, (2) a 1-minute demonstration of the breathing technique, (3) a 15-minute guided practice session, and (4) a 2-minute orientation for independent practice. To minimize potential bias, instructions were delivered using neutral terminology, strictly avoiding persuasive or promotional language.

#### Phase 2: Self-directed practice.

Following training, participants practiced independently twice daily for 14 days. To standardize the practice dosage across sessions, a 3-minute video guide was provided via Google Forms. The video contents resemble face-to-face instruction, but it was shortened and kept only the practice part to capture attention of participants. The video is available on YouTube channel (https://www.youtube.com/watch?v=x0-NKbGzvm4). This format was designed to support consistent implementation while minimizing external supervision and allowing for naturalistic use in daily life. To minimize potential bias, the developer (S.K.T) did not participate in data collection, analysis, or interpretation in this study.

#### Phase 3: Assessment.

Participants were instructed to submit their practice log every time. To initiate the self-directed phase and support protocol adherence, a single LINE message reminder was dispatched by the researcher at the onset of the 14-day study period. This minimal prompt mimics real-world health engagement, where initial orientation or a singular reminder is provided to reinforce autonomy. This reminder was standardized, friendly, and non-coercive (e.g., “As you begin the self-directed phase, please remember to complete your SKT1 session and submit your log daily at your convenience”). Participants were explicitly instructed that they could practice at any time of day. This single-prompt approach reflects an ecologically valid implementation context while ensuring that practice consistency remained primarily self-driven during the remainder of the study.

Psychological measures (stress, anxiety, and resilience) were collected as part of a broader study examining longer-term outcomes of SKT1 practice. Pre-post changes of those outcomes are reported elsewhere and were not the focus of this study. In the present report, baseline scales were used solely as Level-2 predictors of immediate within-session mood changes within the multilevel framework, rather than as primary outcomes.

### Session-level measures

#### Pre-practice negative mood.

Before each SKT1 self-directed practice session, participants rated their current level of negative mood using a single-item numeric rating scale (NRS) ranging from 0 (“no negative feelings”) to 10 (“extremely negative feelings”). Specifically, the scale was intended to capture general mood valence (the overall intensity of negative feeling) rather than discrete or multidimensional emotional states.

#### Post-practice negative mood.

Immediately after each practice session, participants rated their negative mood again using the same single-item 0–10 numeric rating scale. The pre- and post-practice negative mood were recorded and time-stamped via Google Forms.

#### Immediate change score.

An immediate change score was derived by subtracting post-practice negative mood from pre-practice negative mood. Positive values indicated a reduction in negative mood within the session. These session-level data were treated as nested data points within individuals for the subsequent multilevel analyses.

The use of a single-item mood measure was strategic and consistent with best practices in ecological momentary assessment (EMA) and intensive longitudinal research [19], prioritizing momentary mood states over stable trait-level mood. Single-item measures are widely utilized in repeated-measures designs to minimize participant burden, reduce the risk of survey fatigue, and enhance adherence [20]. Research on EMA methodology indicates that single-item mood measures correlate strongly with multi-item scales (*r* > .70) and exhibit comparable sensitivity to rapid momentary fluctuations [21]. However, it is acknowledged that single-item measures lack psychometric depth and cannot capture the multidimensional nature of mood (e.g., distinguishing between sadness, anxiety, or irritability). This trade-off was deemed acceptable given the study’s descriptive focus on immediate, session-level shifts rather than comprehensive mood profiling. In addition to quantitative ratings, participants provided brief qualitative reflections via an open-ended field in the Google Forms; these qualitative data will be analyzed and reported in a separate manuscript.

### Baseline measures

#### Stress.

Perceived stress was assessed using the Srithanya Stress Scale-5 (ST-5) [22], a 5-item self-report scale utilizing a 4-point Likert format. Total scores range from 0 to 15, with higher scores reflecting greater perceived stress. The ST-5 has demonstrated good reliability in prior research (Cronbach’s *α* = .82) [22]. For the present sample, internal consistency was modest (Cronbach’s *α* = .72).

#### Anxiety.

Anxiety symptoms were assessed at baseline using the Generalized Anxiety Disorder Scale-7 (GAD-7) [23], available on Pfizer’s website [24]. This instrument consists of seven items rated on a 4-point Likert scale (total score range: 0–21), where higher scores indicate greater anxiety severity. The GAD-7 is a widely validated instrument with demonstrated criterion, construct, and factorial validity [23]. In the current study, the GAD-7 showed high internal consistency (Cronbach’s *α* = .89).

#### Resilience.

Baseline resilience was measured using the 28-item Thai Resilience Scale [25], rated on a 5-point Likert scale, with higher scores indicating greater resilience. The scale has demonstrated robust content validity (CVI = .86) and reliability in previous research [25]. Internal consistency in the present sample was high (Cronbach’s *α* = .89).

#### Data filtering and dataset construction.

The original sample initially included 28 participants. One participant was excluded due to technical connectivity issues during the self-directed phase, resulting in a final sample of 27 participants. The dataset was structured to ensure a balanced observation window. Participants were instructed to complete 28 sessions; however, as practice was self-directed, adherence varied naturally under minimally prompted, self-directed conditions. To minimize potential bias from over-represented, highly adherent individuals who practiced beyond the protocol [26], only the first 28 sessions from each participant were retained for analysis. This truncation ensured a balanced observation window across the sample, preventing the final multilevel estimates from being disproportionately skewed by a small subset of highly adherent participants.

**Fig 1:**
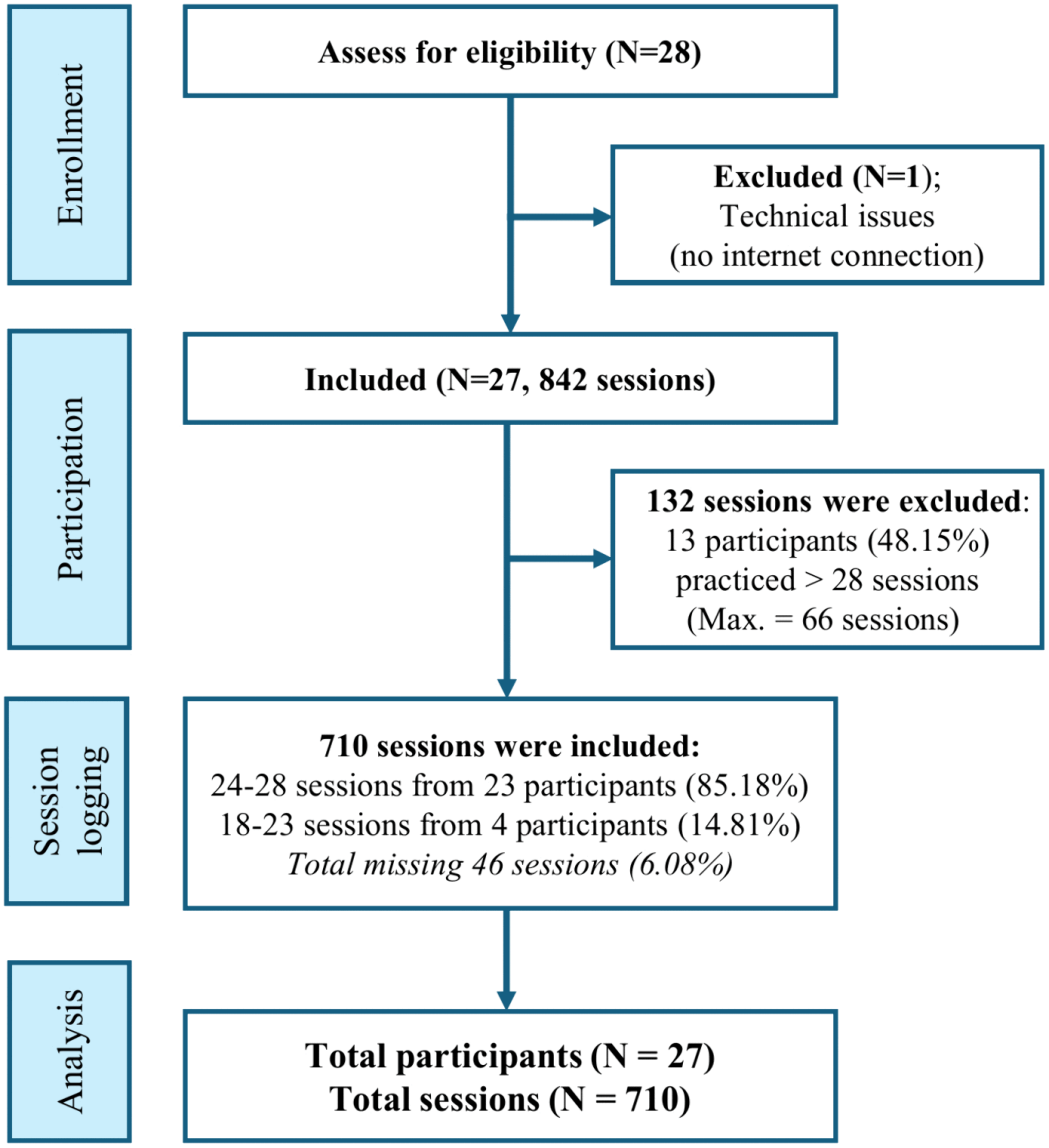
CONSORT diagram. Flow of participants and practice session logs through the study phases.

Accordingly, 132 sessions exceeding the 28-session protocol were excluded, resulting in a final longitudinal dataset of 710 sessions. While 13 participants (48.15%) practiced beyond the requirement, 12 participants completed between 18–27 sessions, resulting in a minimal missing data rate of 6.08%. To appropriately account for this hierarchical structure and the unbalanced nature of the longitudinal observations, Linear Mixed Models (LMM) were utilized for the primary analyses. The participant and session flow is summarized in the CONSORT diagram (Fig 1).

### Ethical considerations

This study was conducted in accordance with the Declaration of Helsinki and received formal approval from the Human Research Ethics Committee of Suranaree University of Technology (COA no. 79/2565, October 17, 2022). Prior to enrollment, all participants provided written informed consent and were explicitly informed of the voluntary nature of their participation, including their right to withdraw at any stage without consequence or penalty. To ensure participant confidentiality, all data were anonymized using unique identification codes. Practice logs were submitted via Google Forms, which was configured not to collect identifying information, such as IP addresses or email identities. Data were stored in secure, password-protected electronic files accessible only to the primary research team. In accordance with privacy standards, only aggregated results are reported in this manuscript to prevent the potential identification of any individual. Furthermore, psychological support resources were made available to all participants throughout the study duration.

### Data analysis

A post-hoc power analysis (G*Power 3.1.9.4) was conducted, yielding a statistical power (1 – *β*) exceeding .99 to detect a medium within-person change (*f* = 0.25, *𝛂* = .05), assuming a 0.34 correlation among repeated measures. While we acknowledge the inherent methodological limitations of post-hoc power estimation compared to a priori calculations—as it can be redundant with *p*-values—this intensive longitudinal design with 710 observations remained highly sensitive to mood fluctuations despite a modest 6.08% missing data rate.

All analyses were conducted via the IBM SPSS 29.0 MIXED procedure. Linear Mixed Models (LMM) served as the primary framework to account for the hierarchical nesting of sessions (Level 1) within participants (Level 2). By modeling these levels simultaneously, LMM appropriately addressed the non-independence of repeated observations. To ensure a clear separation of effects, within-person changes were modeled at Level 1, while between-person differences (e.g., baseline traits) were modeled at Level 2. The LMM framework allowed us to estimate the fixed effect of pre- to post-session change while accounting for the random intercept of each individual, thereby isolating the immediate practice-related mood reductions from stable inter-individual differences. Models utilized Restricted Maximum Likelihood (REML) estimation, which provides unbiased parameter estimates under the Missing At Random (MAR) assumption, offering a robust advantage over traditional ANOVA in handling unbalanced longitudinal datasets.

To determine the optimal error structure, the Diagonal covariance structure was selected. This structure was chosen because it is flexible enough to allow for different levels of variability (variance) at each session while assuming that, after accounting for the individual, the session-specific errors remain independent. In our analysis, this structure provided the superior fit for the data, as evidenced by the lowest Akaike Information Criterion (*AIC*) and Bayesian Information Criterion (*BIC*) compared to alternative models (such as Compound Symmetry or First-Order Autoregressive). This indicates that accounting for session-specific differences in variability was the most effective way to represent the data’s unique patterns.

Seven LMM variants were constructed; the full model—incorporating stress, anxiety, and resilience—demonstrated the superior fit based on model selection criteria. Effect sizes were quantified using Nakagawa et al.’s marginal and conditional *R*^*2*^, alongside Cohen’s *d* [27]. Two complementary *d* indices were computed: paired *d* (primary metric for within-person change) and pooled *d* (for benchmarking against between-person literature). Finally, the Intraclass Correlation Coefficient (*ICC*) quantified the proportion of total variance attributable to between-person differences.

## Results

### Descriptive statistics and practice adherence

A total of 27 participants completed the study, contributing 710 practice sessions for the final analysis. Baseline characteristics and adherence data are summarized in Table 1. The sample was predominantly female (*n* = 19, 70.37%) with a mean age of 20.06 years (*SD* = 1.09). At baseline, participants reported moderate levels of stress (*M* = 10.35, *SD* = 3.13) and anxiety (*M* = 10.47, *SD* = 6.09), with a mean resilience score of 53.18 (*SD* = 11.95).

**Table 1:** Participant characteristics and practice adherence (*N* = 27).

Characteristics	*M (SD)* or *n* (%)	Range
Demographic		
Age (years)	20.06 (1.09)	18-22
Female	19 (70.37%)	—
Baseline mental health		
Resilience	53.18 (11.95)	28-78
Stress	10.35 (3.13)	4-15
Anxiety	10.47 (6.09)	0-20
Practice adherence (Total sessions completed)	26.3 (2.8)	18-28
High adherence	23 (85.19%)	24-28
Moderate adherence	4 (14.81%)	18-23
Session-level Outcomes (*N* = 710)		
Sessions showing negative mood reduction	485 (68.31%)	—
Sessions showing no change	195 (27.46%)	—
Sessions showing increased negative mood	30 (4.22%)	—

Adherence to the 28-session protocol was high. Participants completed an average of 26.3 sessions (*SD* = 2.8; range: 18–28), representing a 93.92% completion rate of the total planned sessions. Most participants (*n* = 23, 85.19%) demonstrated high adherence (≥ 24 sessions), while the remaining 14.81% (*n* = 4) showed moderate adherence (18–23 sessions). No participants demonstrated low adherence or withdrew from the study after the intervention commenced, suggesting favorable feasibility and acceptability of the self-directed SKT1 program.

At the session level (*N* = 710), an immediate reduction in negative mood was observed in 68.31% (*n* = 485) of all practice occasions. Negative mood remained unchanged in 27.46% (*n* = 195) of sessions, while a slight increase in negative mood occurred in only 4.22% (*n* = 30) of sessions.

### Multilevel modeling of SKT1 utility (RQ1–4)

The Intraclass Correlation Coefficient (ICC) was 0.31, suggesting that approximately 31% of the total variance in post-practice negative mood was attributable to stable inter-individual differences, further validating the necessity of the multilevel approach. Fixed effects from the full LMM are presented in Table 2. The overall model demonstrated a strong fit. The marginal *R*^*2*^ (*R*^*2*^_*m*_) was .70, indicating that the fixed effects, specifically practice parameters and baseline psychological traits, explained 70% of the variance in post-practice mood. The conditional *R*^*2*^ (*R*^*2*^_*c*_) was .78, suggesting that the full model, incorporating both fixed and random effects, explained 78% of the total variance.

**Table 2:** Linear mixed model results for post-practice negative mood (Full model).

Parameter	Estimate (*b*)	*SE*	*df*	*t*	*p*	95% CI
Intercept	1.015	1.079	21.64	0.941	.357	[-2.225, 3.256]
**Pre-practice mood (RQ1)**	**0.572**	**0.014**	**522.79**	**42.122**	<.001***	**[0.545, 0.598]**
Session number (RQ2)	−0.004	0.005	277.27	−0.885	.367	[-0.013, 0.005]
Adherence (RQ3)	−0.212	0.384	21.94	−0.552	.587	[-1.008, 0.584]
Baseline resilience (RQ4)	−0.006	0.014	21.37	−0.431	.671	[-0.036, 0.024]
**Baseline stress (RQ4)**	**−0.128**	**0.068**	**21.38**	**−1.877**	**.074†**	**[-0.270, 0.014]**
Baseline anxiety (RQ4)	0.038	0.039	21.35	0.963	.347	[-0.044, 0.119]

*Note: *N* = 27 participants, 710 sessions. ***p < .001, †*p* < .10 (marginal trend).

Model estimated using REML with a diagonal covariance structure

### Immediate changes and temporal stability (RQ1 & RQ2)

The primary objective was to determine the immediate within-session changes of self-directed SKT1. Linear Mixed Model analysis revealed a significant relationship between pre- and post-practice mood levels (*b* = 0.572, *t*(522.79) = 42.12, *p* < .001) (see Table 2), establishing it as a primary predictor of post-session states. In practical terms, this coefficient indicates that for every 1-unit of negative mood reported before practice, only about 0.57 units remained immediately after the session. This reflects an average reduction of approximately 42.8% in negative mood intensity per session (calculated as 1–0.572 = 0.428).

**Fig 2:**
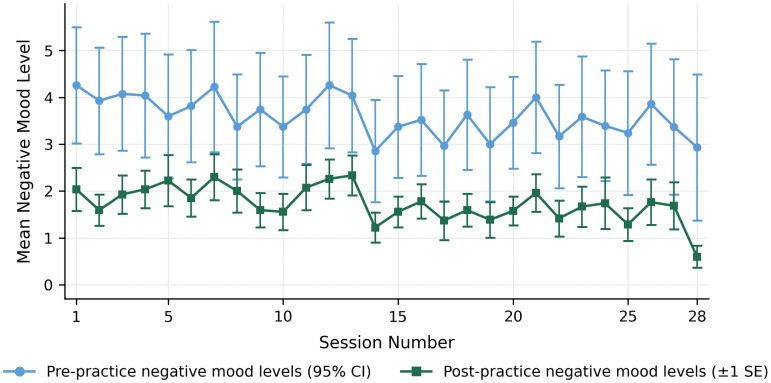
Immediate mood changes associated with SKT1 practice across 28 sessions over 14 days. Error bars represent 95% confidence intervals (CIs).

This reflects a consistent and significant reduction in negative mood, as the post-session scores remained substantially lower than the pre-session baselines regardless of the initial mood level. When coupled with the observed downward shift in mood scores (Fig 2), these results suggest consistent immediate mood reductions across the 710 practice occasions, yielding a large within-person effect size (Paired Cohen’s *d* = 1.00, 95% *CI* [0.91, 1.09]; Pooled *d* = 0.71).

Regarding temporal stability (RQ2), the main effect of session number was non-significant (*p* = .367), indicating immediate mood changes associated with SKT1 remained consistent throughout the 14-day intervention period (28 sessions) (Fig 2).

### The role of adherence and individual differences (RQ3 & RQ4)

For RQ3, practice adherence (total sessions completed) showed no significant association with immediate mood outcomes (*p* = .587). This suggests that the immediate changes associated with SKT1 were experienced consistently across participants, regardless of their total practice frequency within the study window.

**Fig 3:**
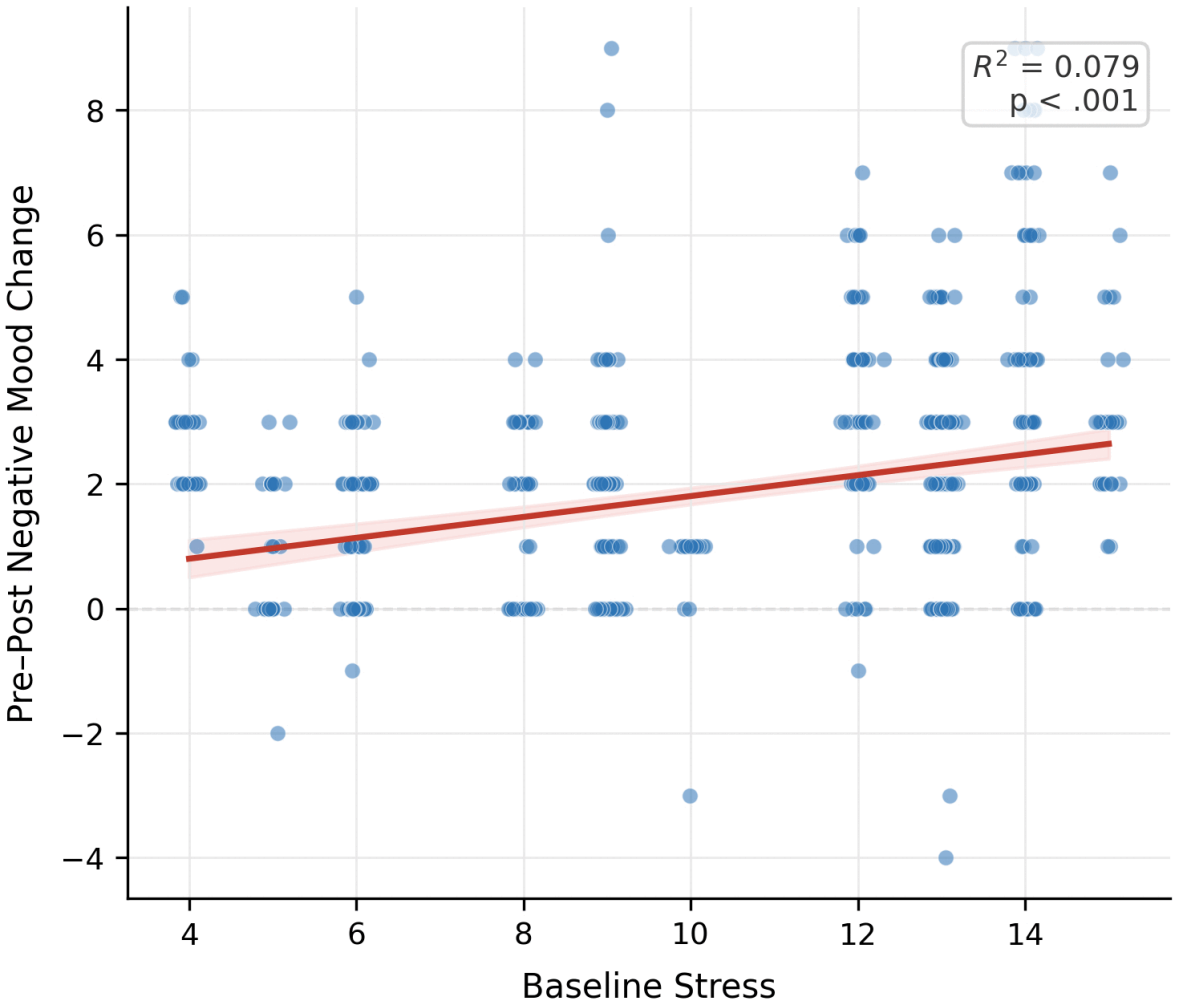
Association between baseline stress and immediate mood reduction. The shaded area represents the 95% confidence interval (CI) of the predicted values. Individual data points represent observed session-level changes.

For RQ4, baseline psychological traits were examined as moderators of immediate practice-related changes. In the full model (Table 2), baseline stress emerged as the strongest association among the tested psychological traits, although this was characterized by a marginal trend (*b* = −0.128, *p* = .074). This suggests that individuals with higher baseline stress levels experienced slightly greater immediate mood reductions (Fig 3; *R*^*2*^ = 0.079).

Supplementary model comparisons (S1 Text) identified a statistical suppression pattern between stress and anxiety. While anxiety alone showed no significant changes (*p* = .735; see S1 Fig), its inclusion in the full model enhanced the predictive precision of baseline stress (increasing the magnitude of the stress coefficient from *b* = −0.059 in the stress-only model to *b* = −0.128 in the full model). This suggests that the association between SKT1 practice and mood reduction may be more pronounced in relation to the physiological arousal associated with perceived stress rather than generalized anxiety. Baseline resilience did not significantly moderate immediate mood changes (*p* = .671; see S2 Fig).

## Discussion

Using a diagonal LMM with 710 sessions nested within 27 participants, four research questions were examined: immediate mood reduction (RQ1), temporal stability (RQ2), the role of practice adherence (RQ3), and the association between baseline stress levels and immediate outcomes (RQ4). The findings indicate a consistent association between self-directed SKT1 practice and immediate within-session mood reductions. It is important to emphasize that this research was designed as a preliminary pilot exploration (proof-of-concept). While the number of participants is relatively small (*N* = 27), the intensive longitudinal design—yielding 710 nested observations—provides a granular and robust look at immediate within-person mood dynamics that might be overlooked in larger, aggregate group-level studies.

### Immediate negative mood reduction (RQ1)

SKT1 sessions were associated with immediate reductions in negative mood (*b* = 0.572, *p* < .001), with a large within-person effect size (Paired Cohen’s *d* = 1.00). This finding aligns with emerging evidence suggesting that brief, physiologically-oriented interventions can reliably be associated with immediate shifts in mood states. Our results are in accordance with those of Balban et al. [28], who demonstrated that exhale-focused cyclic sighing produced greater immediate mood reduction than mindfulness meditation, theoretically involving proposed vagal involvement. Nevertheless, the magnitude of this effect (*d* = 1.00) may be partly influenced by expectancy bias or social desirability, as participants were aware of the study’s focus on mood reduction. These factors may have systematically inflated the effect estimates, necessitating a cautious interpretation of the intervention’s potency.

Furthermore, the potential for social desirability bias was significantly mitigated by the multidisciplinary nature of the sample. As participants were recruited from various faculties (e.g., Engineering, Public Health) where the researcher had no direct instructional role, the risk of students providing favorable responses to please an instructor was minimized. Combined with the participants’ lack of familiarity with the technique developer, these factors strengthen the validity of the reported mood improvements.

Several safeguards were in place. Crucially, participants had no knowledge of Dr. SKT or her role as the developer of the technique. This social distance from the developer, combined with the fact that she was not present during the study, suggests that the large effect size is more likely a reflection of the technique’s immediate impact rather than an attempt to please a high-profile developer.

Our findings support the “bottom-up” approach of SKT1. By prioritizing structured breathing and rhythmic movement, the procedure is theorized to bypass the heavy cognitive load required by top-down mindfulness practices, making it a potentially accessible tool for acute mood management in a self-directed format. This immediate shift could be related to supporting physiological balance, providing a physiological anchor that disrupts negative mood cycles [16,17]. However, as noted by Shao et al. [29], while these acute changes are notable, they may reflect transient shifts in state mood rather than immediate alterations in autonomic baselines. This distinction is critical for university-based interventions, where the goal is often to provide readily available relief from academic-related distress without requiring the extensive training associated with traditional contemplative techniques.

### Temporal stability across sessions (RQ2)

The extent of negative mood reduction remained remarkably stable across 28 sessions (*b* = −0.004, *p* = .367). This lack of a temporal trend suggests the absence of both a ceiling effect and habituation over the 14-day period. Unlike complex cognitive interventions that may require an extended “learning curve” before mood reductions are realized, the procedural simplicity of SKT1 appeared to allow students to derive consistent changes from the first session through the last. This suggests that the technique’s potential utility does not rely on cumulative mastery but rather on the immediate feedback associated with each practice. Consequently, SKT1 may serve as a potential “mood-focused first-aid” tool that maintains its utility with repeated use, offering a potential predictable mood management strategy that students in this setting could exploratively deploy at any point in the semester without fear of diminishing returns. This aligns with digital meditation research showing sustained changes across hundreds of sessions [30] and reinforces the potential of SKT1 as a sustainable resource for stress management in academic settings.

### Adherence and dose-response (RQ3)

The adherence level was not significantly associated with immediate mood changes (p = .587), suggesting that the immediate reductions associated with SKT1 may operate independently of the cumulative practice dose. This suggests that the proposed physiological pathways, such as those theorized to involve autonomic balance, may function as acute responses that do not depend on the total frequency of practice within the study period. While adherence is often linked to longer-term neuroplasticity and trait-level changes [31], our findings suggest that even students with ‘moderate’ or inconsistent adherence can derive significant per-session mood reductions. This flexibility is particularly relevant for university populations, as the practice can be integrated into varying daily schedules. Unlike mindfulness programs that may require sustained commitment to achieve detectable results, SKT1 appears to offer fewer barriers to entry with immediate reinforcement. From a public health perspective, this is encouraging; it implies that SKT1 can serve as a flexible resource for students who struggle with consistency, providing immediate mood management whenever it is practiced. However, it must be acknowledged that the high adherence rate (94%) was likely bolstered by a single LINE message reminder sent by the researcher at the beginning of the study phase. While this supports the protocol’s preliminary feasibility, it may not reflect purely naturalistic engagement in the absence of external prompting. Future trials should evaluate whether such consistency is maintained without researcher-led reminders.

### Baseline psychological moderators (RQ4)

A key finding was the identification of a statistical suppression pattern between baseline stress and anxiety. While neither variable reached conventional significance independently, the inclusion of anxiety as a suppressor enhanced the predictive precision of baseline stress (increasing the magnitude of the coefficient from *b* = −0.059 to *b* = −0.128, *p* = .074). This suggests that baseline stress may have a more pronounced association with the observed mood changes once the overlapping variance with generalized anxiety is statistically controlled. This finding implies that SKT1 practice may be more associated with shifts in the acute ‘tension’ component of distress rather than the broader, more stable cognitive patterns associated with trait anxiety.

Furthermore, the non-significant associations for anxiety and resilience (see S1 Fig and S2 Fig) reinforce the broad applicability of SKT1 within the study context. The technique appears consistently useful for students regardless of their baseline psychological hardiness or pre-existing anxiety levels. This lack of moderation is significant; it suggests that SKT1 does not require a specific psychological ‘readiness’ to be associated with mood reductions. Consequently, the results support its exploratory role as a preliminary mood support tool within this specific university context, suggesting that its utility was consistent across individuals with varying levels of psychological resources within this sample.

### Limitations and future directions

Several limitations apply to the present study. First, the small sample size limits the generalizability of the findings to the entire university population. As an exploratory pilot study, the results should be interpreted with caution and primarily as a demonstration of preliminary feasibility. Future studies with larger, randomized samples are required to confirm these preliminary observations and to establish the broader efficacy of self-directed SKT1 practice across more diverse demographic groups.

Second, the absence of a control condition fundamentally precludes causal inference. Consequently, mood changes may partly reflect regression to the mean, expectancy changes, or general relaxation rather than SKT1-specific mechanisms, requiring cautious interpretation. Our findings document associations between SKT1 practice and immediate mood changes in a naturalistic context; they do not establish causation. Future controlled studies with randomized comparison conditions are necessary to isolate the specific potential utility of SKT1.

Third, the voluntary nature of the study likely introduced a self-selection bias, where students already interested in or motivated by mind-body practices were more likely to enroll. This may have resulted in a sample that is more responsive to SKT1 than the general university population, potentially overestimating the technique’s broader appeal and utility.

Fourth, although SKT1 is a brief somatic technique, the potential for adverse psychological effects from meditation, as reported in 10–20% of practitioners [32], cannot be dismissed. Future utility trials must incorporate formal monitoring for adverse events to establish a comprehensive safety profile, particularly for vulnerable student populations.

Fifth, the sample was small, drawn from a single institution, and predominantly female (70.37%), which restricts the generalizability of the findings. Existing literature suggests that women may engage more with emotion-focused coping and report greater mood reductions following mindfulness-based interventions [33,34]. Additionally, the study was conducted at a single Thai university, restricting its applicability to other cultural or age groups. Future research should prioritize gender-balanced samples and cross-cultural replication to validate these preliminary associations.

Sixth, participants’ awareness of being monitored may have induced a Hawthorne effect, potentially creating a ‘social desirability’ bias where participants reported more positive mood changes to align with perceived researcher expectations; this may have inflated the observed mood reductions.

Seventh, while the single-item mood measure is consistent with ecological momentary assessment (EMA) best practices to minimize participant burden [19,35], it lacks psychometric depth and is susceptible to measurement error. Specifically, the scale captures general mood valence only and cannot distinguish between specific emotional states (e.g., sadness vs. irritability). Furthermore, we acknowledge that the simplicity of this single-item measure may have contributed to an inflation of the observed effect sizes. Consequently, these preliminary results should be interpreted as indicative of momentary mood fluctuations rather than clinically meaningful therapeutic change.

Eighth, the use of a single LINE message reminder at the onset for participants who had not yet completed their daily sessions likely contributed to the notably high adherence rate (94%). While this proactive monitoring ensured data completeness, it suggests that maintaining such consistency in a purely naturalistic setting without external prompts may be more challenging, with implications for the study’s ecological validity.

Finally, one author (S.K.T.) developed the SKT1 technique and is affiliated with the non-profit meditation center (SMAEM SKT Meditation Healing Exercise Center). While steps were taken to ensure independence, this affiliation could theoretically introduce an allegiance bias, potentially influencing the framing or interpretation of the findings. Independent validation by researchers with no institutional ties to the program is recommended to ensure impartiality in future utility trials.

## Conclusion

The findings of this preliminary study suggest that self-directed SKT1 meditation is associated with consistent, immediate reductions in negative mood among university students. These observed changes remained stable across 28 sessions over a 14-day period and were not contingent on practice frequency or baseline psychological profiles. The application of Linear Mixed Modeling with a diagonal covariance structure facilitated a nuanced and accurate understanding of the associations between brief contemplative practices and mood changes within naturalistic settings, appropriately accounting for both the hierarchical structure of intensive longitudinal data and session-specific variance patterns. Given the exploratory nature of this study, the observed preliminary feasibility within this minimally prompted context suggests that SKT1 may serve as a potential mood management resource specifically for university students in similar contexts. In conclusion, these preliminary pilot findings offer a proof-of-concept for the use of self-directed SKT1 in university settings. Although further research is needed to validate these results in larger populations, the current study highlights the potential of brief, physiologically-oriented practices as an accessible resource for student mood management.

## Supporting information

S1 Data Minimal data set. Underlying data used for all analyses in this study. Available at Mendeley Data (doi:10.17632/gnm2j5tb34.1).
(XLSX)

S1 Text Supplementary methodological text and tables. Contains Supplementary Text S1 (Statistical Suppression Pattern in Stress and Anxiety Moderation), Supplementary Table S1 (Sequential LMM Model Comparison and Fixed Effects), and Supplementary Table S2 (Correlation Matrix of Baseline Variables).
(DOCX)

S1 Fig Association between baseline anxiety and immediate mood changes. The shaded area represents the 95% confidence interval (CI) of the predicted values.
(TIF)

S2 Fig Association between baseline resilience and immediate mood changes. The shaded area represents the 95% confidence interval (CI) of the predicted values.
(TIF)
