# Supplementary material for: Immediate mood changes and practice adherence during a self-directed SKT1 meditation program in university students: An intensive longitudinal study
Source: PLoS One. 2026 May 29;21(5):e0350556. doi: 10.1371/journal.pone.0350556 (PMC13220996; doi:10.1371/journal.pone.0350556)
Supplement: S1 Text — Contains Supplementary Text S1 (Statistical Suppression Pattern in Stress and Anxiety Moderation), Supplementary Table S1 (Sequential LMM Model Comparison and Fixed Effects), and Supplementary Table S2 (Correlation Matrix of Baseline Variables). (DOCX) [file pone.0350556.s002.docx]

**Supporting information**

**Title: Immediate mood changes and practice adherence during a self-directed SKT1 meditation program in university students: An intensive longitudinal study**

**Supplementary Text S1. Statistical Suppression Pattern in Stress and Anxiety Moderation**

Sequential model comparison identified a statistical suppression pattern between baseline stress and anxiety. In the best-fitting bivariate model (Model 6, *AIC* = 2066.039), baseline stress showed a non-significant association with immediate mood reduction (*b* = –0.059, *p* = .168). However, when baseline anxiety was included in the full model (Model 1, *AIC* = 2075.765), the magnitude of the stress coefficient more than doubled (*b* = –0.128, *p* = .074).

This phenomenon indicates that baseline anxiety acted as a suppressor variable, removing irrelevant variance from the stress predictor and enhancing its predictive precision. This pattern suggests that the mood-management potential of SKT1 are more specifically associated with the physiological arousal component of perceived stress once the shared variance with generalized anxiety is statistically controlled.

**Supplementary Table S1. Sequential LMM Model Comparison and Fixed Effects (Models 1–7)**

**Note:** Models are strictly sorted by AIC (Best Fit First). This arrangement highlights the statistical superiority of the stress-only model while allowing for comparison of coefficient changes in the full model.

| **Model** | **Description** | ***AIC*** | **Δ*AIC*** | **Baseline Stress (*b*)** | ***SE*** | ***p*** | **Baseline Anxiety (*b*)** | ***SE*** | ***p*** |
| --- | --- | --- | --- | --- | --- | --- | --- | --- | --- |
| **Model 6** | **Stress Only (Best Fit)** | **2066.039** | **0** | **–0.059** | **0.042** | **.168** | **-** | **-** | **-** |
| Model 5 | Composite Distress | 2068.966 | 2.927 | - | - | - | - | - | - |
| Model 7 | Anxiety Only | 2069.167 | 3.128 | - | - |  | –0.008 | 0.022 | .735 |
| Model 4 | Baseline (Neither) | 2070.607 | 4.568 | - | - | - | - | - | - |
| Model 1 | Full Model | 2075.765 | 9.726 | –0.128 | 0.068 | .074† | +0.034 | 0.038 | .347 |

**Note**: All models were estimated using Restricted Maximum Likelihood (REML) with a Diagonal covariance structure. **Δ***AIC* is calculated relative to the best-fitting model (Model 6). †*p* < .10 (marginal trend).

**Supplementary Table S2. Correlation Matrix of Baseline Variables**

| **Variable** | **1** | **2** | **3** | **4** |
| --- | --- | --- | --- | --- |
| 1. Pre-practice negative mood | 1.000 |  |  |  |
| 3. Baseline Stress | **.263***** | 1.000 |  |  |
| 4. Baseline Anxiety | **.273***** | **.789***** | 1.000 |  |
| 2. Baseline Resilience | **–.202***** | **–.452***** | **–.620***** | 1.000 |

*Note: Spearman’s *ρ* correlations. *N* = 710 sessions from 27 participants. ****p < .001.*

The strong correlation between Stress and Anxiety (*ρ* = .789) accounts for the suppression effect observed in Model 1.

**S1 Fig. Association between baseline anxiety and immediate mood changes.** *The shaded area represents the 95% confidence interval (CI) of the predicted values. Individual data points represent observed session-level changes.*

Scatter plot illustrating the relationship between participants' baseline anxiety scores (GAD-7) and the mean reduction in negative mood across sessions. (Note: The association was non-significant, *p* = .735).

**S2 Fig. Association between baseline resilience and immediate mood changes.** *The shaded area represents the 95% confidence interval (CI) of the predicted values. Individual data points represent observed session-level changes.*

Scatter plot illustrating the relationship between baseline resilience (BRS) and mood changes. (Note: The association was non-significant, *p* = .671).
